# Effects of Separation of Cows and Calves on Reproductive Performance and Animal Welfare in Tropical Beef Cattle

**DOI:** 10.3390/ani9050223

**Published:** 2019-05-08

**Authors:** Agustín Orihuela, Carlos S. Galina

**Affiliations:** 1 Facultad de Ciencias Agropecuarias, Universidad Autónoma del Estado de Morelos, Av. Universidad 1001, Colonia Chamilpa, Cuernavaca 62210, Morelos, Mexico; 2 Departamento de Reproducción, Facultad de Medicina Veterinaria y Zootecnia, Universidad Nacional Autónoma de México, Ciudad Universitaria, Ciudad de México 04510, Mexico; cgalina@unam.mx

**Keywords:** wellbeing, reproduction, Zebu, stress

## Abstract

**Simple Summary:**

This review summarizes knowledge on the separation of cows and calves and its effect on the onset of ovarian activity and animal welfare, principally in tropical beef cattle. After a brief introduction establishing the importance of calf separation to induce ovarian activity in their mothers and the welfare aspects that are jeopardized during this process, subsequent sections describe different strategies used for the separation of cows and calves, highlighting the effect of each method on beef cattle reproductive performance and welfare considerations of both cow and calf; conclusions and practical implications.

**Abstract:**

Nursing a calf suppresses postpartum ovarian activity prolonging the period of anestrus. Diverse methods are used to reduce the effect of suckling; the most popular, restricted suckling, reduces the number of encounters mother-calf. Temporal weaning of the calf for periods of 24 h, 48 h, or even 72 h also suppress the effect of suckling and is commonly applied to cow-calf operations in the tropics. Early weaning of the calf, usually three to five months after birth, is a practice gaining popularity over the traditional system of weaning at seven months. Furthermore, the use of nose-flaps in the calf to avoid suckling is a common procedure in South America. Finally, weaning during the first week after calving is an established method to reduce postpartum anestrus. The objective of the present review is to discuss the effects of these methods on the reproductive performance of beef cattle and their animal welfare implications.

## 1. Introduction

The late resumption of postpartum ovarian activity in Zebu cattle is seen as a major hurdle for achieving a calving interval between 12 months and 14 months [1]. An early review on the subject [2] pointed to the two primary causes for the delay in the onset of ovarian activity following parturition: one was the effect of suckling, and the other, the loss of body weight even prior to calving [3]. Different methods for the temporary separation of mother and calf during lactation have been developed to reduce the calving conception interval, which together with better feeding programs have helped achieve the desired results.

It is well known that suckling stimulus and the mere presence of the calf, are among the most important factors affecting the duration of anestrus postpartum in beef cattle [4,5,6]. Thus, a considerable amount of research has been vested in this area [6,7], showing that various systems of separation have a beneficial effect on the early restoration of postpartum ovarian activity. However, this practice may induce different levels of stress, defined as an “environmental effect on an individual which over-taxes its control systems and reduces its fitness” [8]. Pérez et al. [9] found that separation of the offspring has a stressful effect on both the mothers and the offspring as shown by the display of behaviors associated with stress, like vocalizations and locomotor activity, increase in cortisol concentration, loss of weight, and close proximity to fence-line, among others. Nowadays, society demands fair treatment of animals [10], so the productive advantages of shortening the parturition-first ovulation interval and reducing the calving interval, must be considered together with the need to subject the animals to the minimum possible stress [11].

Some of the environmental stressors that cows and calves face during temporal weaning and even months after their reintegration include an increase in udder pressure, severance of the calf–cow bond, a shift in eating habits of the calf and adaptation to new social companions [12]. Suckling interruption results in the accumulation of milk in the udder, leading to udder engorgement and increased tension provoking discomfort and pain [13]. Some of the behavioral signs associated with stress reported in dairy cows due to this condition include vocalization for several days and altered standing-lying behavior [14,15] even following the omission of a single milking event [16]. In addition, Bertulat et al. [17] and Odensten et al. [18] found that fecal and blood cortisol concentrations increased after milking suspension. The severance of the calf–cow bond refers to the denied social and physical contact between them. In general, this bond is stronger in young calves. Weary et al. [19], found that younger animals are more socially dependent upon the dam. Accordingly, Pérez-Torres et al. [20] found that 25-day-old calves were observed within <10 m from the fence line regardless of the separation period, a pattern not observed in 45-day-old calves, suggesting a greater independence from their mothers. Calves also must change their eating habits, moving from a liquid to a solid diet; from suckling to grassing or concentrate consumption. To reduce potential stress, the introduction of concentrates early on from a few days of age, is recommended to encourage intake [21]. Calves also need to adapt to new social companions, as normally weaned calves are moved to a different pen or pasture with other calves. The regrouping and mixing of unfamiliar animals might lead to a tenfold increase in agonistic interactions observed during the hours following the event. Many studies report behavioral and physiological consequences of repeated social changes that could reflect social stress in beef cattle [22,23,24].

Several weaning strategies used in tropical beef cattle leading to different reproductive performance and stress responses in cows and calves, are described below. All these techniques are applied with very variable results, which are also affected by body condition, nutrition, parity, and age, among other factors (for reviews see Galina et al. [25] and Burns et al. [26]).

## 2. Weaning Methods

### 2.1. Restricted Suckling Periods

In this method the calf is allowed to suckle only for short periods of time, usually not more than 30 min per day [7] for periods of 72 h or until the day of weaning [27].

#### 2.1.1. Reproductive Performance

Since the earlier studies of Mukasa-Mugerwa [28], considerable research has been published on the effect of suckling on the resumption of ovarian activity in cattle raised under tropical conditions. Margerison et al. [29] determined that suckling once daily did not reduce reproductive performance compared to artificial rearing, and concluded that suckling cows twice daily increases total milk production at the expense of reducing body weight in early lactation. Cows nursing their own calves have inferior reproductive performance compared to those nursing other calves or not nursing at all with their calves reared artificially. Similarly, Sahn et al. [30] concluded that restricted suckling could be considered more advantageous than calf removal and artificial rearing for managing cows and calves. In the last ten years, the use of different hormonal protocols to facilitate estrous detection, considered a major hurdle in cattle raised under tropical conditions, has been the subject of considerable research effort (for reviews see Bo et al. [31]; Baruselli et al. [32]). It is outside the scope of this review to analyze all the different combinations of protocols, either to regulate the estrous cycle [33], or to induce a fertile estrus in anestrous animals [34,35]. The protocols are applied either in the presence of the calf or utilizing the different calf separation methods described in the present review. 

Work has been carried out specifically addressing the effect of environmental–nutritional interactions on calf performance. Dixon et al. [36] carried out an interesting experiment where cows were subjected to two nutritional regimes were compared in the dry and the wet seasons. Calf growth was not affected by nutritional regime during the early dry season (April–July), but was slower in the mid dry season (July–September) for cows on the poor nutritional diet. They concluded that cows on the poor nutritional regime mobilized enough additional body reserves to maintain milk production during the July–September period, but not during the harsh conditions of April–July. Also, Rodríguez and Segura [37] working in Mexico found that once-daily suckling increased pregnancy rates of zebu-cross cows without lowering growth performance of the calf. In addition, pregnancy rate was higher in supplemented than in control cows, with or without suckling. These experiments underline the complex interactions between season of the year, supplementation strategies and an array of calf separation techniques. The economic tradeoff between dietary supplementation, milk yield, and reproductive performance have yet to be determined in restricted suckled cows.

#### 2.1.2. Cows and Calves’ Stress Response

There are several variants of the restricted suckling strategy. For example, calves can be allowed to suckle for one or two 30 min periods per day, or calves and cows might be kept together during night hours. During these contact periods calves suckle at libitum and freely interact with their mothers. During the separation periods, cows can pasture while their calves remain enclosed and may receive some concentrate. The mother young bond is disrupted for periods of about 23 h or only 12 h alternated with 12 h of coexistence, respectively during these practices.

Calves seem to adjust within a couple of days [27] to the process that might last from three days, starting around 45 days postpartum (dpp) and up to several weeks, until weaning. Acevedo et al. [27] found that body temperature, respiration, and heart rates showed a significant decrease following the rise observed the first day of treatment. It can be speculated that as calves quickly learn that will be reunited with their dams after a short period, they may regulate their consumption accordingly. Cows also get used to the procedure of foraging actively during the separation periods. During contact, their udders are empty and their social needs with their calves can be satisfied.

Older calves seem to be less distressed during the procedure than younger calves. Several studies have shown that total suckling duration per day decreases with age of the calf [38,39,40]. This could be explained by a lower incidence of suckling bunts in older animals [38,41], resulting in less suckling episodes missed, together with the possibility of compensating the reduction in milk consumption with the ingestion of solid food. In addition, exploration and play become more frequent from ages 1 month to 6 months [38], suggesting improved wellbeing of older calves. Little published research exists on the behavior of calves during group restricted suckling, for instance it is not known how frequently calves suckle their dams during the sessions of restricted suckling.

Although separation from their mothers is stressful, keeping the calves in a familiar environment with other pen mates may allow them to adjust to separation from their mother more easily than moving them to an unfamiliar pen or putting them with new pen mates. Proponents of these methods argue that allowing calves to remain in a familiar environment following maternal separation reduces stress [42], as calves in novel environments display escape behaviors, search for social partners [43], and suffer fear [44]. In addition, calves display more positive social interactions with familiar calves during separation [45], which results in a calming effect [46]. Even though some increase in labor is required for the daily management of the herd, techniques like accustoming young animals to brief separations from their mother, may reduce the impact of separation at weaning [47].

In conclusion, cows seem to be less affected by the removal of their calves than the calves themselves, regardless of the treatment applied [27,48].

### 2.2. Temporal Weaning

This method involves the suppression of suckling for periods from 12 h to 96 h [49,50] between 25 days and 90 days postpartum (dpp) [25,51], with complete removal of the offspring from the dam with such variants as allowing certain levels of visual, auditory or odor contact between the cows and calves as opposed to complete separation [52,53,54].

#### 2.2.1. Reproductive Performance

Several studies have shown the beneficial effects on reproductive performance when the calves are separated from their mothers in periods ranging from 24 h to 72 h (for review see Galina et al. [25]). This procedure may be accompanied by supplementary feed, various hormonal regimes, visual contact or even monitoring the metabolic status of the animal during gestation, parturition, and early postpartum. The age of the animal, breed and number of calvings, coupled with different combinations of nutritional regimens or other environmental factors, make it difficult to draw conclusions. For example, Soto-Camargo et al. [55,56] demonstrated that supplementary feed in early postpartum in cows calving for the first time, influences weight gain in the dams but not their reproductive performance. The same group, [57] supplemented prior and after calving obtained an increase in weight gain of the dams but again, not in reproductive performance. Delgado et al. [58] calculated the odds of finding a cycling or pregnant cow at 120 dpp in the dry tropics of México. These parameters improved with increasing body condition score. Cows calving in the dry or rainy season had less possibilities of cycling or pregnancy than those calving during the windy and rainy season. First calving cows had the worst performance, no differences were found among multiparous cows. Rubio et al. [59] concluded that stocking rate did not affect follicular population dynamics of Brahman cows, but body condition score dictated the number and categories of follicles present regardless of the stocking rate utilized. These studies tend to suggest that supplementary feeding in the postpartum period has little or no effect on reproductive performance.

However, monitoring body weight changes during the late gestation seems to be a valuable tool to predict early reproductive success postpartum. Díaz et al. [60] reported that the resumption of ovarian function, as characterized by the metabolic profile and body condition, was not affected by the combination of calf separation and progestogen treatment. Moreover, in further experiments, Díaz et al. [60] found that animals with adequate metabolic conditions during the latter part of gestation have a better chance of pregnancy regardless of the time postpartum when the reproductive program starts. Comparing two locations in the dry tropics of Mexico and Costa Rica, Díaz et al. [61] found a different pattern in body weight equilibrium prior to and after calving but a common feature for stability was the adaptability to variations in the temperature humidity index. Jolly et al. [62] in Australia concluded that low body condition score at calving was associated with prolonged postpartum anestrus. However, when the dams maintained their live weight, ovarian cyclicity resumed within 50 days. These studies call attention to the fact that the environmental conditions in a given location dictate the success of reproductive performance. Thus, a comparison between studies seem to be of little use when the indicators for metabolic and environmental stability are not specified.

The monitoring of ovarian activity following a drug synchronization regime has been adequately studied [63]. There is a considerable number of publications on the subject by Baruselli et al. [64]. In a broader sense, drug treatments can be helpful if the animals have adequate body condition, but risks of impaired follicular growth and reduced fertility can be found if animals are borderline in this respect prior to or at the time of treatment [65,66].

#### 2.2.2. Cows and Calves’ Stress Response

Acevedo et al. [27] compared the effect of restricted suckling and temporal weaning on physiological and behavioral stress parameters in Zebu cattle finding that calves in the restricted suckling treatment had lower levels of serum cortisol, less locomotion activity and vocalizations, and spent more time eating and lying down, in comparison with those in the temporal weaned treatment, suggesting that brief periods of suckling or social contact (i.e., 30 min per day during three days of separation) may reduce anxiety. Also, Hötzel et al. [67] observed that calves subjected to temporary mother deprivation, showed an earlier return to baseline values compared to controls.

All physiological and behavioral parameters indicative of stress in calves decrease over time, especially after the second day of separation, suggesting adaptation to separation from their dams [27]. This agrees with several studies in different ruminant species showing that stress responses of offspring and their dams did not go beyond 72 h after separation [68,69].

Previous studies [20,27,70] agree that cow-calf removal for 24 h results in fewer behavioral and physiological indices of distress compared to a 72-h separation. Furthermore, Price et al. [71], Acevedo et al. [27] and Pérez-Torres et al. [20], demonstrated that in general cows are less affected than their young, according to the results showing that mothers as well as their young exhibit reuniting behaviors (locomotion, calling) following separation although the duration and intensity of efforts to reunite tend to be greater in offspring than their mothers.

Pressure in the udder is an important source of pain, leading to an increase in cortisol concentration with respect to pre- calf separation. This is more notorious after a 72-h separation, particularly in cows during early postpartum around the time of the lactation peak (25 dpp). Milk accumulation might favor the development of bacteria and can lead to udder health problems [72,73]. Also, temporal weaning induces significant increases in walking, butting, urinating, and vocalizing [70], suggesting psychological stress like that reported when weaning beef calves [69,74].

In one experiment, Pérez-Torres et al. [20], three groups of animals were separated from their dams, one for 24 h, another for 48, and a third for 72 h. A clear pattern in the distance of the 25 dpp cows from the fence-line was observed, with more cows close to the fence-line (<10 m) during the first 24 h of separation, with the strength of attraction progressively weakening with time. However, at 45 dpp the highest percent of the cows were seen at <20 m from the fence-line, which can be the result of a weaker cow-calf bond at a more advanced postpartum age. The same authors found that more calves vocalized at 24 h and 48 h in comparison to 72 h, but with lower values in older calves. They also found that most stress indicators in cows and calves decreased after 48 h of separation, with increases in cortisol concentration and weight losses inversely proportional to the duration cow-calf separation [20].

Price et al. [71] also found that behavioral signs of stress in calves lasted for three days during a fence-line separation study. They also noticed synchronized grazing and resting behaviors during the three days of fence-line separation, with most of the calves grazing together away from the fence and then returning to stand or lie down near this barrier. The distance traveled increased over days following separation. These findings are consistent with the hypothesis that social bonds between calves are strengthened during separation and that calf groups tend to behave as a unit. Veissier and LeNeindre [75] showed that weaned calves gather together and have more social encounters with one another than similar-age calves that remained with their mothers. This cohesion in recently separated calves might be a way to cope with the recently broken mother-young relation.

In general, longer separations or repeated calf withdrawals result in higher cow pregnancy rates with reduced periods of anestrus postpartum [76] pending on the time when the intervention is performed. However, longer separations bring the risk of severance of the mother–young bond, the cows not accepting to nurse their calves at all, with the consequent problem of early weaning, artificial rearing or fostering mothers, augmenting considerable costs and labor. It is logical to think that age postpartum and parity at least would be factors to consider in the breaking up of the mother-young bond.

The management practice of temporal weaning applied to calves early in life (i.e., 25–45 days old), can prove impractical under extensive range conditions due to the cost of supplementary fodder for calves because at this age they depend on milk or milk substitutes to survive, in addition to the continuous management required and concerns for the health of the animal. Furthermore, stress in cows and calves might be especially high when calves are separated early in life from their mothers, as the bond between them is still very tight [77]. Young calves particularly are more socially dependent upon their dam [78]. Hopster et al. [68] also hypothesized that when the removal of the calf occurs at a later age, the accompanying sensory information fades, and stress may be reduced.

The lack of protective behavior during the separation periods, that cows provide for their calves may further increase losses by predation in cattle raised under extensive conditions [48,79]. Long periods of separation might also affect certain learning processes occurring during mother–young cohabitation. Moreover, even though it has not been studied in beef cattle, research in dairy cows [80] has demonstrated the long-term effects of early maternal deprivation, in terms of forming less sociable animals when adults, together with effects on behavior, stress reactivity, and the ability to cope with different challenges.

Older calves display fewer signs of stress than younger animals [20]. These authors found that whilst most of the 25-day-old calves were observed within <10 m from the fence-line regardless of the separation period, this pattern was not observed in older calves. In addition, less weight loss has been found in older than younger calves. Firstly, because young animals have lower reserves of body fat [81] and secondly, because older calves are able to ingest and assimilate solid food during separation, which reduces not only their weight loss, but also the likelihood of becoming hunger and reach starvation conditions.

### 2.3. Precocious or Early Weaning

The method consists of weaning the calf during the first week of birth. This strategy requires supplementary feeding for the young and involves an early break in the mother-calf bond.

#### 2.3.1. Reproductive Performance

It is obvious that weaning calves early in the postpartum has a beneficial effect on the reproductive performance of the dam (for review see Chenoweth [82]; Galina et al. [25] analyzed the consequences of different suckling systems for reproductive activity in tropical cattle and commented on the controversy surrounding which method is the most appropriate for reproductive performance and the wellbeing of the dam and offspring. Jolly et al. [83] concluded that early weaning had a greater effect than postpartum nutritional supplements on the resumption of ovarian activity in first-lactation heifers with moderate nutrition.

#### 2.3.2. Cows and Calves’ Stress Response

Beef calves are frequently weaned from their dams when they are between 180 days and 220 days of age [42], while natural weaning occurs around 10 months for Zebu type cattle [40]. Early weaning is defined as separating calves from their dams at less than 180 days of age, with a range from as early as 45 days of age but averaging about 70–90 days of lactation [84,85,86]. This technique is most commonly practiced when feed is scarce or where breeding females are at risk from reproductive failure because high nutrient requirements are not met by a poor diet.

From the productive point of view, early weaning has resulted in heavier calves at the time when normal weaning would have occurred (i.e., >180 days of age), which could be interpreted as better wellbeing. Cows are less stressed than calves, and behavioral changes induced by weaning are greater in multiparous than primiparous cows [87].

The most common technique is to move calves from pasture to a feeding pen beyond the reach of sight, smell, and hearing of the dams, or to separate calves and dams into adjacent pastures (fence-line), reducing stress caused by separation by allowing them to socialize while preventing nursing.

When mutually strongly bonded mothers and young are separated prematurely, bouts of reuniting behavior such as locomotion (searching) and vocal signaling are performed by both and interspersed by periods of energy-conserving depression [88].

In dairy cows, Weary and Chua [78] found that dams vocalized more after separation from their calves at four days that at 6 h or 1 dpp. Furthermore, increased vocalization when separated from their calves at four days post-partum is accompanied by an increase in the amount of visible eye-white displayed [89]. Young animals are supposed to cope better than older animals when confronting painful or stressful situations [90]. However, in the case of weaning, it is important to consider that in younger ruminants the bond with their mothers is initially very strong, but declines with increasing age of the young and decreasing milk production [91]. Possibly for this same reason, calf separation is more effective in inducing cyclic activity in postpartum cows with younger calves.

### 2.4. Fence-Line

This method comprises the removal of the calf to a contiguous pasture where mother and calf can see, smell and hear each other, but nursing is not possible [71].

#### 2.4.1. Reproductive Performance

Quesada et al. [53] compared cows in anestrus previously synchronized whose calves were separated for 48 h without visual or olfactory contact with those whose calves were able to suckle through a fence-line. The former group showed better reproductive response. Webb et al. [54] used the same approach but separating the calves for 72 h with only visual contact and reported an increase in the number of animals cycling. Bolaños et al. [92] found that pharmacologically inducing estrous treatment could enhance the percentage of postpartum cows displaying behavioral estrus whether or not the calf was separated for 48 h but were unable to achieve cyclicity in about 50% of those cows treated. These conflicting results could be explained by the experiments of Pérez-Torres et al. [51] who compared the reproductive response of separation for 24 h, 48 h, and 72 h. The variability of the response at 48 h seems to be the cause of differences in relation to testing reproductive efficiency. In effect, calf separation for 72 h seems to be the most logical way to mimic the duration of the proestrus phase and thereby achieve more acceptable growth of the follicular milieu. In fact, Mondragón et al. [65] and Xavier et al. [66] found that follicular diameter can be impaired if suckling continues as compared to calf separation with a possible detrimental effect on fertility.

#### 2.4.2. Cows and Calves’ Stress Response

The fence-line was essentially developed to reduce stress, as a technique that isolates the termination of suckling from the social separation of the calf-dam pair by allowing social contact but not suckling. Furthermore, this technique can be combined with the other methods described for suckling interruption.

Nicol [93] Stookey et al. [69] and Price et al. [71] compared post-weaning growth and behavior of calves that had either fence-line contact with their dams or were abruptly and completely separated at weaning. They found that fence-line contact calves gained more weight than their abruptly separated counterparts and that the well-being of the calves and cows was improved when allowing some social contact between them. However, Solano et al. [70] found the opposite to be true. Nonetheless, most studies agree that during the first days of fence-line separation calves spend more than half the time near the barrier separating them from their mothers, suggesting a high motivation to reunite [71,94].

### 2.5. Nose-Flaps

This method uses plastic devices that prevent calves from suckling but allow them to graze, eat and drink water while remaining in contact with their mothers [94].

#### 2.5.1. Reproductive Performance

Dill et al. [95] investigated the differences between weaning rates and technologies adopted by farmers in cow-calf production systems in Brazil. Through interviews and cluster analysis, they found that the highest adoption rate was in the use of nose flaps in calves with no apparent reproductive differences compared to other methods, the main factor being an association between higher weaning rates and greater adoption of various technologies. Enriquez et al. [94] used a two-step weaning strategy utilizing nose flaps during the breeding period. Their observations agreed with previous data [84,85] confirming that the application of this technique could have a beneficial effect on reproductive performance. However, an early review [6], suggests that not only suckling but the physical presence of the calf impairs the early restoration of ovarian activity. Effectively, the author concludes that only when viewed from the perspective of production agriculture, weaning does become a problem. Solutions require further appreciation of both biological and non-biological limitations.

#### 2.5.2. Cows and Calves’ Stress Response

As in fence-line, this technique isolates the termination of suckling from the social separation of the calf–dam pair by allowing social contact but not suckling and can also be combined with the other methods of cow-calf separation.

Compared to total abrupt separation the use of nose-flaps reduces bawling and walking, while increasing time spent eating [96]. However, it is also reported [96,97] that calves fitted with nose-flaps had lower average daily weight gains and their welfare may have been jeopardized due to their frustration when attempting to nurse several times and not being able to after placement of the device [98]. On the other hand, the only response observed in cows after fitting the nose-flaps was a small increase in vocalizations, probably in response to the increase in udder pressure or in response to the calls of the calves, which significantly increase their vocalization in the days after nose flaps are fitted [67,94].

## 3. Conclusions

The method of restricted sucking appears to be the technique more attune to the natural conditions of most mammals including humans [99]. The stressful effect on the dam and the young remains to be clarified but the fact that animals adapt reasonably quickly to situations which are temporal [94] shows promising results.

Separated calves should be grouped or penned based on body size, with familiar members to discourage undesirable aggressive encounters, reduce stress and promote a social buffering effect. Familiar physical environments should be favored, as should some social contact amongst calves and cows (i.e., fence-line). Calves should eat as soon as possible after separation. Starter rations and water should always be available. Creep feeding some days before separation could be recommended to get the animals familiar with solid food. Early weaned calves that have not been acclimated previously to concentrate-based feeds can be fed hay. Social facilitation of peers, familiar with the food may encourage consumption.

While carrying out this review, the authors became aware of a lack of information on the effects of cow and calf separation strategies on reproductive performance and animal welfare in tropical beef cattle, particularly in Zebu type cattle. Research on this topic must be developed to establish practical recommendations specific to these animals and thus avoid the misplaced use of information generated in other breeds and environments. 
